# Hysterectomies Are Associated with an Increased Risk of Depression: A Population-Based Cohort Study

**DOI:** 10.3390/jcm7100366

**Published:** 2018-10-18

**Authors:** Tomor Harnod, Weishan Chen, Jen-Hung Wang, Shinn-Zong Lin, Dah-Ching Ding

**Affiliations:** 1 Department of Neurosurgery, Hualien Tzu Chi Hospital, Buddhist Tzu Chi Medical Foundation, Tzu Chi University, Hualien 970, Taiwan; tomorha@yahoo.com.tw (T.H.); shinnzong@yahoo.com.tw (S.-Z.L.); 2 Management Office for Health Data, China Medical University Hospital, Taichung 404, Taiwan; sandy8121985@gmail.com; 3 College of Medicine, China Medical University, Taichung 404, Taiwan; 4 Department of Research, Hualien Tzu Chi Hospital, Buddhist Tzu Chi Medical Foundation, Tzu Chi University, Hualien 970, Taiwan; jenhungwang2011@gmail.com; 5 Department of Obstetrics and Gynecology, Hualien Tzu Chi Hospital, Buddhist Tzu Chi Medical Foundation, and Tzu Chi University, Hualien 970, Taiwan; 6 Institute of Medical Sciences, Tzu Chi University, Hualien 970, Taiwan

**Keywords:** hysterectomy, depression, hormone therapy, oophorectomy, cohort

## Abstract

Using the National Health Insurance Research Database of Taiwan, we investigated whether undergoing a hysterectomy increases the risk of depression. A total of 7872 patients aged 30–49 years who underwent a hysterectomy from 2000 to 2013 were enrolled as the hysterectomy group. The comparison group was randomly selected from women who had never undergone a hysterectomy and was four times the size of the hysterectomy group. We calculated adjusted hazard ratios and 95% confidence intervals (CIs) for depression [The International Classification of Diseases, Ninth Revision, Clinical Modification (ICD-9-CM) codes 296.2, 296.3, 300.4, 311] in these cohorts after adjusting for age, comorbidities, oophorectomy, and hormone therapy. The overall incidence of depression was 1.02 and 0.66 per 100 person-years in the hysterectomy and comparison cohorts, respectively, yielding an adjusted hazard ratio of 1.35 (95% CI = 1.22–1.50) for depression risk. When we stratified patients by age, comorbidities, oophorectomy, and hormone use, hysterectomy increased the risk of depression. Hysterectomy, oophorectomy, and post-surgery hormone use were associated with an increased risk of depression when they occurred alone, but conferred a greater risk if they were considered jointly. Hysterectomy would be a predisposing factor for increased risk of subsequent depression. Our findings provide vital information for patients, clinicians, and the government for improving the treatment strategy in the future.

## 1. Introduction

Hysterectomy is a common gynecological surgery performed to remove the uterus in women with uterine myoma, endometriosis, uterine prolapse, genital cancers, and other benign conditions [1,2]. The prevalence of hysterectomy ranges from 5.1 to 5.8 per 1000 women in the United States and from 2.68 to 3.03 per 1000 women in Taiwan [1,2,3]. Most hysterectomies are performed in premenopausal adult women to treat benign conditions [3,4] with the primary goal of improving their quality of life rather than lifespan [4,5]. In addition to detailing the mostly positive outcomes of the procedure for treating gynecological symptoms [5], the psychological and sexual functioning outcomes should also be considered to enable patients to make a fully informed decision to undergo a hysterectomy.

It has been commonly assumed that undergoing a hysterectomy could cause adverse effects on psychological well-being that might be associated with an increased risk of depression. Some studies have suggested that hysterectomy may lead to decreased psychological well-being, whereas others have not found evidence of an association between this type of surgery and depression [6,7]. Reports have suggested that undergoing a hysterectomy might reduce symptoms of depression and anxiety and increase general well-being [6,8,9,10]. One study suggested that post-hysterectomy symptoms might be a continuation of patients’ pre-surgery depression, anxiety, or hostility [11]. Another study discovered that women who underwent a hysterectomy (regardless of whether or not they underwent a bilateral oophorectomy) had a higher risk of developing new depressive symptoms in the postoperative follow-up period that could not be explained by the patients’ lifestyles or socioeconomic factors [12].

Women with depression were reported to fare worse 24 months after undergoing a hysterectomy than women who either had depression alone, or neither underwent a hysterectomy nor had depression [13]. A prospective study indicated that women who underwent a hysterectomy may have a risk of psychiatric morbidity [14]. Another prospective study determined that depression scores were reduced five years after hysterectomy compared with pre-hysterectomy scores among women who underwent hysterectomies [15]. Persson et al. indicated that general psychological well-being was equally improved 12 months after undergoing either a subtotal hysterectomy or a total hysterectomy [9]. These various and inconsistent results based on the extant literature have failed to clearly identify the relationship between undergoing a hysterectomy and postoperative depression. However, worse sexual function after hysterectomy, emotional problems associated with poorer body image, and higher stress after surgery are possible risk factors for post-hysterectomy depressive disorder [10]. Most studies were performed in Western countries and may not be applicable to Asian societies. In order to clarify and fill the clinical gap in the literature, we studied the association between hysterectomy and subsequent development of depression.

Taiwan is located in Eastern Asia and the government launched the National Health Insurance (NHI) program covering more than 99% of Taiwan’s population in 1995 [16,17]. Therefore, we designed this study using the NHI database in Taiwan to explore the correlation between undergoing a hysterectomy and subsequent depression.

## 2. Materials and Methods

### 2.1. Data Source

The National Health Research Institute released the National Health Insurance Research Database (NHIRD) for research purposes. The Longitudinal Health Insurance Database 2000 (LHID2000) is a database based on data in the NHIRD. The LHID2000 randomly selected 1 million patients from the NHIRD, and included the patients’ personal information and insurance claims. To protect patient privacy, identification numbers were recoded. This study was approved by the Institutional Review Board (IRB) of China Medical University and Hospital (CMUH) Research Ethics Committee (REC) (IRB permit number: CMUH-104-REC2-115).

### 2.2. Sampled Participants

**Figure 1:**
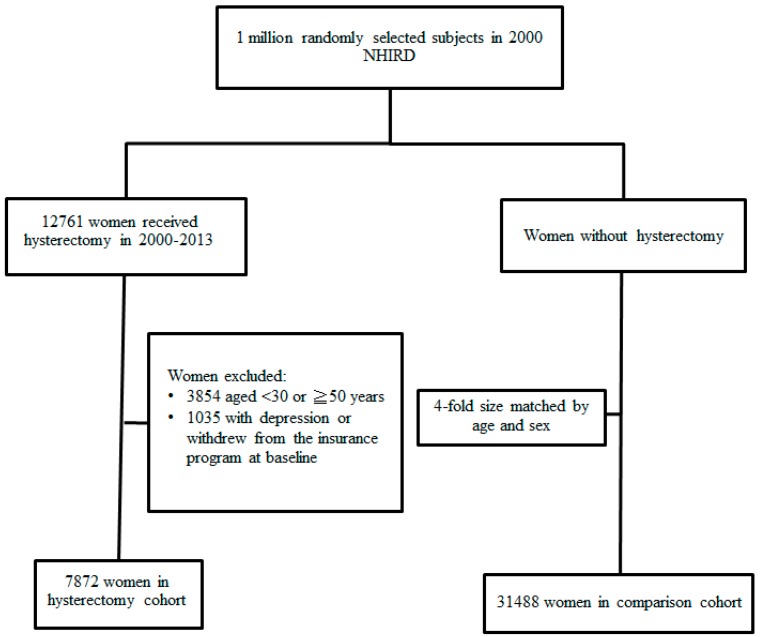
Flow chart of the process for establishing the hysterectomy cohort and controls using the National Health Insurance Research Database.

We enrolled women aged 30–49 years who underwent a hysterectomy with or without an oophorectomy from 2000 to 2013 as the hysterectomy group for this study. The date of surgery was the index date. We excluded patients who had been diagnosed with depression (ICD-9-CM codes 296.2, 296.3, 300.4, 311) or withdrew from the insurance program before the index date. The comparison group for this study was randomly selected from women who had not undergone a hysterectomy and was four-fold in size matched with the hysterectomy group by sex, age, and index year. The selection process is illustrated in Figure 1.

### 2.3. Outcomes, Relevant Variables, and Comorbidities

The outcome of this study was depression, and the endpoint was the date when a patient received a diagnosis of depression, withdrew from insurance, or the end of 2013. Person-years were the sum of the follow-up time for each individual, and the follow-up time was the period from the index date to the endpoint. The comorbidities controlled for in this study were anxiety (ICD-9-CM code 300), cancer (ICD-9-CM codes 140–208), stroke (ICD-9-CM codes 430–438), and coronary artery disease (CAD; ICD-9-CM codes 410 to 413, 414.01 to 414.05, 414.8, and 414.9). We considered whether the patients who underwent hysterectomies also underwent an oophorectomy and used hormone replacement after the hysterectomy. 

### 2.4. Statistical Analysis

To analyze the demographics of the hysterectomy group and the comparison group, we used the Chi-squared test for category variables and the *t*-test for continuous variables. The incidence rate of depression was calculated by person-years. The hazard ratio (HR) and 95% confidence interval (CI) of the two groups were estimated using univariate and multivariate Cox proportional hazards regression models. The variables analyzed in the multivariate model were age, oophorectomy, hormone use, and comorbidities of anxiety, cancer, stroke, and CAD. We used the Kaplan–Meier’s method to describe the depression-free probability of the two groups and tested the difference between the two groups using the log-rank test. The data analysis for this study was performed using SAS statistical software (Version 9.4 for Windows; SAS Institute, Inc., Cary, NC, USA). The level of statistical significance was set as *p* < 0.05. 

## 3. Results

The study cohort consisted of 7872 patients who had undergone a hysterectomy, and the comparison cohort consisted of 31,488 patients who had not. As evident in Table 1, the distribution in age was similar between cohorts. The hysterectomy cohort had a higher proportion of individuals who had undergone an oophorectomy as well as all comorbidities. The mean follow-up times for patients who had undergone a hysterectomy and the comparison cohort were 7.0 and 7.6 years, respectively (Table 1). 

**Figure 2:**
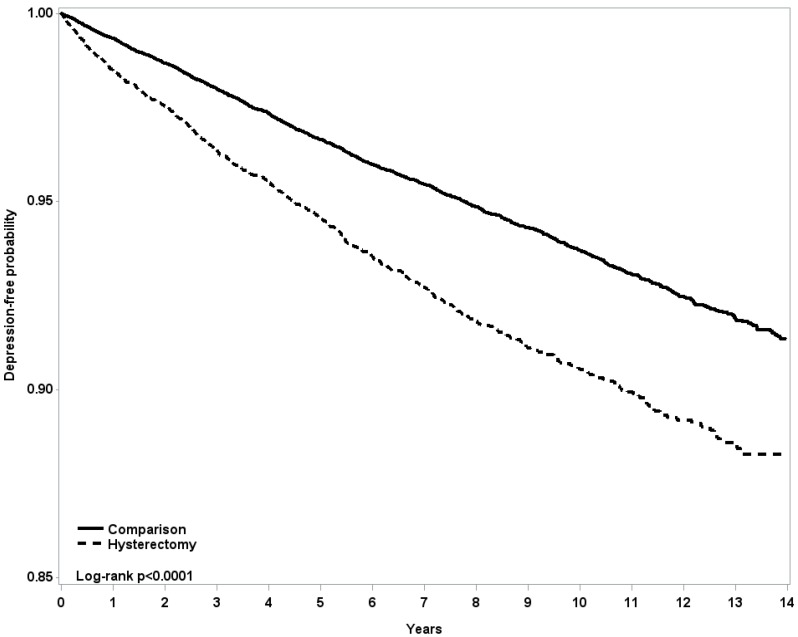
The Kaplan-Meier method was used to measure the depression-free probability for women who underwent a hysterectomy (dashed line) and controls (solid line) by the end of the follow-up period.

Figure 2 shows the results of applying the Kaplan–Meier method to indicate that the hysterectomy cohort had lower depression-free probability than the comparison cohort during the entire follow-up period. Table 2 presents the findings that the incidence of depression was higher in the hysterectomy cohort than in the comparison cohort (1.02 vs. 0.66 per 100 person-years), and the adjusted HR of depression was 1.35 (95% CI = 1.22–1.50). When we stratified patients by different ages, the patients in the hysterectomy cohort exhibited higher risks for depression in the group aged 30–39 years (adjusted HR = 1.57, 95% CI = 1.24–1.99) and in the group aged 40–49 years (adjusted HR = 1.30, 95% CI = 1.16–1.47) than the in the comparison group (Table 2).

Then, we divided our patients into four subgroups based on whether they underwent hysterectomy or oophorectomy (Table 3). Compared to women who never underwent hysterectomy and oophorectomy, women underwent hysterectomy and oophorectomy had the highest risk of depression (adjusted HR = 1.46, 95% CI = 1.23–1.72). Women who underwent hysterectomy and not oophorectomy had the second highest risk of depression (adjusted HR = 1.34, 95% CI = 1.20–1.50), but there was no significant difference for the non-hysterectomy and oophorectomy groups (Table 3). 

We also analyzed the joint effects of hysterectomy and hormone replacement on the risk of depression in patients. Compared to non-hysterectomy and non-hormone used women, the risk of depression for hysterectomy women with hormone use was 1.63 (95% CI = 1.40–1.91), for hysterectomy ones without hormone use was 1.42 (95% CI = 1.26–1.60), and for non-hysterectomy women with hormone use was 1.34 (95% CI = 1.19–1.52) (Table 4).

In Table 5, we depict the risk of depression for women with hysterectomy, oophorectomy, and hormone use. Compared to women without hysterectomy, without oophorectomy, and without hormone use, the risk of depression for hysterectomy women with oophorectomy and non-hormone use was 1.37 (95% CI = 1.07–1.74) and for hysterectomy women who used hormone but not received oophorectomy was 1.48 (95% CI = 1.23–1.79). Hysterectomy women with oophorectomy and hormone use had the highest increased risk of depression (adjusted HR = 1.99, 95% CI = 1.61–2.47) (Table 5).

For comorbidities, we further analyzed whether hysterectomy women have an increased risk of depression if they had comorbidities of anxiety, stroke, and CAD (Table 6). Compared to non-hysterectomy women without these comorbidities, the risk of depression for hysterectomy women without these comorbidities was 1.46 (95% CI = 1.29–1.67). Hysterectomy women with only one of the comorbidities had a higher risk of depression (only anxiety: adjusted HR = 3.68, 95% CI = 3.09–4.40; only stroke: adjusted HR = 2.92, 95% CI = 1.83–4.67; only CAD: adjusted HR = 3.34, 95% CI = 2.07–5.41). Hysterectomy women with more comorbidities had a much higher risk of depression, especially in hysterectomy women with stroke and CAD (adjusted HR = 9.78, 95% CI = 4.38–21.8), and hysterectomy women with all these comorbidities (adjusted HR = 7.25, 95% CI = 3.01–17.5) (Table 6).

## 4. Discussion

Taiwanese women who underwent a hysterectomy in the 30–49-year-old age group had a higher post-hysterectomy depression risk compared with the general population. This was true for the patients regardless of whether they had also undergone an oophorectomy or had one of the comorbidities of anxiety, cancer, stroke, or CAD. The results of this observational study revealed that hysterectomy, oophorectomy, and post-surgery hormone use are associated with an increased risk of depression when they occur alone, but confer a greater risk if they are experienced jointly. The same findings were observed for the comorbidities. Our findings and the results of another study [12] suggest that once a hysterectomy is performed on a woman, early-premenopausal hormone therapy might not be beneficial for improving her psychological health.

Luteinizing hormone (LH) closely regulates ovarian function, and any abnormal control of LH pulsatile release has major implications for all levels of the hypothalamic-pituitary-ovarian (HPO) axis. In LH regulation, estrogen is not only a steroidal hormone released from the ovary for controlling the estrous or menstrual cycle in women, but also has a role in numerous other systems such as the neuroendocrine, skeletal, and immune systems in both sexes [18]. Disruption of negative feedback regulation in the HPO axis due to a hysterectomy might cause LH and estrogen dysregulation problems. In some women older than 45 years who had received uterine artery embolization (UAE) as a treatment for fibroid tumors, amenorrhea caused by reduce ovarian blood flow from ovarian ligaments leads to premature ovarian failure [19]. Another study suggested that major depressive disorder was correlated with the disruption of LH pulsatility in the follicular phase of the menstrual cycle [20]. Therefore, disruptions in LH and estrogen regulation after a hysterectomy might be the major mechanism that causes these patients to experience increased depression risk. Women underwent oophorectomy in addition to hysterectomy had higher anxiety-related scores, lower sexual variable scores, and poorer partner relationships [8]. These operations had a joint effect on the increased risk of depression. Although ovarian conservation at hysterectomy might be beneficial for a lowering the risk of developing subsequent depression, the results of ours and other studies revealed that both women underwent a hysterectomy with ovarian conservation and those underwent a hysterectomy with oophorectomy would have an increased risk of depression [12,14]. The results imply that hysterectomy itself would be a predisposing factor for developing depression, with or without oophorectomy.

Estradiol therapy slows the clearance of serotonin by activating estrogen receptors that decrease serotonin clearance. Ovariectomized rats receiving estradiol therapy exhibited antidepressant effects similar to those from serotonin supplementation [21]. Among aging women, decreased estrogen levels significantly decrease the brain’s serotonin levels, thereby increasing the incidence and symptoms of depression. This dysfunction should be corrected through hormone therapy in daily practice [22,23]. Oophorectomy results in premenopausal ovarian insufficiency and multiple health risks in patients, including menopausal symptoms, decreased bone density, early progression of cardiovascular disease, dry eye syndrome, and psychological comorbidities that may include depression, anxiety, and cognitive decline. Bove et al. demonstrated that early surgical menopause was associated with Alzheimer Disease neuropathology, increasing neuritic plaques [24]. However, definite biological interactions with oophorectomy, hormone replacement, and possible comorbidities in patients remain unclear. The decrease in estrogen receptor affinity was assumed to play a role in certain mechanisms of estrogen dysregulation associated with aging and psychological comorbidities beyond oophorectomy [18]. In this study, we discovered a risk of 1.02 per 100 person-years for developing depression among women who underwent a hysterectomy at ages 30–49 years. Decades of aging had caused a high risk of estrogen receptor dysfunction, and correlated depression to accumulate at middle age. Our results revealed that hormone therapy had no significant effect on the reduction of depression risk following a hysterectomy and anxiety did interact with hysterectomy for developing depression. Post-hysterectomy anxiety, stress, change in women’s identity and sense of femininity, poorer sexual function, and subjective gynecological symptoms after surgery might interact together to affect this important dimension of women’s lives and lead to depression [10]. Due to that depression after hysterectomy directly increases the care burden on patients’ families, society, and health care systems. Additional studies are necessary to confirm this hypothesis in Taiwan and evaluate whether it is globally applicable.

This study included a nationwide population-based sample with little risk of recall and selection bias; thus, as with similar studies using NHIRD data [17,25,26], our findings are valuable for Taiwanese patients and clinicians and can be used as a reference for other countries with a heritage similar to that of Taiwan. However, this study has several limitations. First, we could not directly contact the study patients because their identities were anonymized in the NHIRD. The pathologies that prompted our patients to undergo hysterectomies were unknown. Various benign and malignant pathologies would likely result in different levels of risk for developing subsequent depression in the cohorts. We intentionally enrolled study patients aged less than 50 years old because the average menopause age in Taiwan is 49.5 years [27]; however, patients’ menopausal statuses were not available or analyzed in this NHIRD study. Second, our cohort did not include women who were older than 50 years because hysterectomies are rarely performed in that age group in Taiwan. Thus, fewer data were available for statistical analysis. Older patients have a relatively high risk of cardiovascular disorders after undergoing a hysterectomy according to the findings of a study that also used NHIRD data [26]. These cardiovascular comorbidities after hysterectomy might possibly result in a higher risk of depression in older women [26]. Third, although the NHI program performs thorough quarterly reviews to ensure that the files are accurate and false claims are heavily sanctioned, miscoding may have nevertheless occurred in the NHIRD. After considering the aforementioned limitations, our results indicated that the sample size was sufficient to statistically demonstrate the subsequent depression risk in patients who had undergone a hysterectomy.

## 5. Conclusions

Hysterectomy with oophorectomy and hormone use jointly affect the increase in depression risk. However, hysterectomy alone increases the risk of subsequent depression in patients, regardless of whether or not they also underwent oophorectomy, with or without hormone replacement, and with or without various comorbidities. Our findings provide vital information for patients, clinicians, and the government for improving depression treatment strategies in the future.

## Figures and Tables

**Table 1:** Baseline characteristics in women with and without hysterectomy.

	Hysterectomy (*n* = 7872)	Comparison (*n* = 31,488)	*p*-Value
	*N* (%)	*N* (%)	
Age, years			
30–39	1400 (17.8)	5600 (17.8)	1.0000
40–49	6472 (82.2)	25,888 (82.2)	
Mean (SD)	43.1 (4.0)	43.1 (4.0)	1.0000
Follow-up duration (years)			
Mean (SD)	7.0 (4.1)	7.6 (4.1)	<0.0001
Oophorectomy	1963 (24.9)	1146 (3.6)	<0.0001
Hormone use	2170 (27.6)	3920 (12.5)	<0.0001
Comorbidity, *n* (%)			
Anxiety	1473 (18.7)	4293 (13.6)	<0.0001
Cancer	280 (3.6)	412 (1.3)	<0.0001
Stroke	282 (3.6)	813 (2.6)	<0.0001
CAD	316 (4.0)	780 (2.5)	<0.0001

CAD: coronary artery disease.

**Table 2:** Risk of depression in women with hysterectomy compared to comparison group.

	*N*	Event	PY	Rate	Crude		Adjusted *	
					HR (95% CI)	*p*-Value	HR (95% CI)	*p*-Value
All								
Comparison group	31,488	1566	238,597	0.66	1 (reference)		1 (reference)	
Hysterectomy	7872	566	55,234	1.02	1.56 (1.41–1.71)	<0.0001	1.35 (1.22–1.50)	<0.0001
Age 30–39								
Comparison group	5600	275	46,844	0.59	1 (reference)		1 (reference)	
Hysterectomy	1400	111	10,718	1.04	1.75 (1.41–2.19)	<0.0001	1.57 (1.24–1.99)	0.0002
Age 40–49								
Comparison group	25,888	1291	191,753	0.67	1 (reference)		1 (reference)	
Hysterectomy	6472	455	44,517	1.02	1.51 (1.36–1.69)	<0.0001	1.30 (1.16–1.47)	<0.0001

PY: person-years; Rate: per 100 PY; HR: hazard ratio; CI: confidence interval; * Model was adjusted for age, oophorectomy, hormone use, and comorbidities listed in Table 1.

**Table 3:** Risk of depression in women underwent hysterectomy or oophorectomy.

Hysterectomy	Oophorectomy	*N*	Event	PY	Rate	Crude		Adjusted *	
						HR (95% CI)	*p*-Value	HR (95% CI)	*p*-Value
No	No	30,342	1509	230,667	0.65	1 (reference)		1 (reference)	
No	Yes	1146	57	7931	0.72	1.09 (0.84–1.42)	0.51	1.00 (0.77–1.30)	0.99
Yes	No	5909	404	40,453	1.00	1.52 (1.36–1.69)	<0.0001	1.34 (1.20–1.50)	<0.0001
Yes	Yes	1963	162	14,781	1.10	1.67 (1.42–1.97)	<0.0001	1.46 (1.23–1.72)	<0.0001

PY: person-years; Rate: per 100 PY; HR: hazard ratio; CI: confidence interval; * Model was adjusted for age, hormone use, and comorbidities listed in Table 1.

**Table 4:** Risk of depression in women among joint hysterectomy and hormone therapy.

Hysterectomy	Use Hormone	*N*	Event	PY	Rate	Crude		Adjusted *	
						HR (95% CI)	*p*-Value	HR (95% CI)	*p*-Value
No	No	27,568	1244	204,231	0.61	1 (reference)		1 (reference)	
No	Yes	3920	322	34,366	0.94	1.56 (1.38–1.76)	<0.0001	1.34 (1.19–1.52)	<0.0001
Yes	No	5702	355	37,167	0.96	1.55 (1.38–1.75)	<0.0001	1.42 (1.26–1.60)	<0.0001
Yes	Yes	2170	211	18,068	1.17	1.93 (1.67–2.24)	<0.0001	1.63 (1.40–1.91)	<0.0001

PY: person-years; Rate: per 100 PY; HR: hazard ratio; CI: confidence interval; * Model was adjusted for age, hormone use, and comorbidities listed in Table 1.

**Table 5:** Risk of depression in women underwent hysterectomy, oophorectomy, and hormone therapy.

Hysterectomy	Oophorectomy	Use Hormone	*N*	Event	PY	Rate	Crude		Adjusted *	
							HR (95% CI)	*p*-Value	HR (95% CI)	*p*-Value
No	No	No	26,673	1207	198,240	0.61	1 (reference)		1 (reference)	
No	Yes	No	895	37	5991	0.62	1.01 (0.73–1.40)	0.96	0.97 (0.70–1.35)	0.85
No	No	Yes	3669	302	32,427	0.93	1.55 (1.36–1.76)	<0.0001	1.34 (1.18–1.53)	<0.0001
No	Yes	Yes	251	20	1939	1.03	1.70 (1.09–2.64)	0.02	1.40 (0.90–2.18)	0.14
Yes	No	No	4566	284	29,500	0.96	1.57 (1.38–1.78)	<0.0001	1.45 (1.27–1.65)	<0.0001
Yes	Yes	No	1136	71	7667	0.93	1.51 (1.19–1.92)	0.0008	1.37 (1.07–1.74)	0.01
Yes	No	Yes	1343	120	10,953	1.10	1.81 (1.50–2.19)	<0.0001	1.48 (1.23–1.79)	<0.0001
Yes	Yes	Yes	827	91	7115	1.28	2.12 (1.72–2.63)	<0.0001	1.99 (1.61–2.47)	<0.0001

PY: person-years; Rate: per 100 PY; HR: hazard ratio; CI: confidence interval; * Model was adjusted for age and comorbidities listed in Table 1.

**Table 6:** Joint effect for depression among joint hysterectomy, anxiety, stroke, and CAD.

Hysterectomy	Anxiety	Stroke	CAD	*N*	Event	PY	Rate	Crude		Adjusted *	
								HR (95% CI)	*p*-Value	HR (95% CI)	*p*-Value
No	No	No	No	26,306	1027	20,4752	0.50	1 (reference)		1 (reference)	
No	Yes	No	No	3669	388	22,946	1.69	3.35 (2.98–3.77)	<0.0001	3.29 (2.92–3.70)	<0.0001
No	No	Yes	No	481	35	3753	0.93	1.86 (1.33–2.60)	0.0003	1.81 (1.29–2.54)	0.0005
No	No	No	Yes	376	26	2793	0.93	1.85 (1.26–2.73)	0.002	1.84 (1.24–2.71)	0.002
No	Yes	Yes	No	252	41	1799	2.28	4.53 (3.32–6.19)	<0.0001	4.32 (3.16–5.91)	<0.0001
No	Yes	No	Yes	324	39	1986	1.96	3.88 (2.82–5.34)	<0.0001	3.71 (2.69–5.12)	<0.0001
No	No	Yes	Yes	32	2	238	0.84	1.67 (0.42–6.69)	0.47	1.69 (0.42–6.75)	0.46
No	Yes	Yes	Yes	48	8	329	2.43	4.83 (2.41–9.68)	<0.0001	4.42 (2.20–8.87)	<0.0001
Yes	No	No	No	6085	344	44,119	0.78	1.55 (1.37–1.75)	<0.0001	1.46 (1.29–1.67)	<0.0001
Yes	Yes	No	No	1228	149	7426	2.01	3.97 (3.35–4.72)	<0.0001	3.68 (3.09–4.40)	<0.0001
Yes	No	Yes	No	155	18	1133	1.59	3.16 (1.98–5.04)	<0.0001	2.92 (1.83–4.67)	<0.0001
Yes	No	No	Yes	142	17	953	1.78	3.55 (2.20–5.74)	<0.0001	3.34 (2.07–5.41)	<0.0001
Yes	Yes	Yes	No	88	9	567	1.59	3.15 (1.63–6.07)	0.0006	2.93 (1.52–5.65)	0.001
Yes	Yes	No	Yes	135	18	789	2.28	4.50 (2.83–7.18)	<0.0001	4.19 (2.63–6.70)	<0.0001
Yes	No	Yes	Yes	17	6	119	5.04	10.0 (4.50–22.4)	<0.0001	9.78 (4.38–21.8)	<0.0001
Yes	Yes	Yes	Yes	22	5	129	3.88	7.65 (3.18–18.4)	<0.0001	7.25 (3.01–17.5)	<0.0001

PY: person-years; Rate: per 100 PY; HR: hazard ratio; CI: confidence interval; * Model was adjusted for age, oophorectomy, hormone use, and cancer.
